# Association between longer hospitalization and development of *de novo* donor specific antibodies in simultaneous liver–kidney transplant recipients

**DOI:** 10.1080/0886022X.2019.1705338

**Published:** 2019-12-26

**Authors:** Masahiko Yazawa, Orsolya Cseprekal, Ryan A. Helmick, Manish Talwar, Vasanthi Balaraman, Pradeep S. B. Podila, Sallyanne Fossey, Sanjaya K. Satapathy, James D. Eason, Miklos Z. Molnar

**Affiliations:** a James D. Eason Transplant Institute, Methodist University Hospital, Memphis, TN, USA; b Division of Transplant Surgery, Department of Surgery, University of Tennessee Health Science Center, Memphis, TN, USA; c Division of Nephrology and Hypertension, Department of Internal Medicine, St. Marianna University School of Medicine, Kawasaki, Japan; d Department of Transplantation and Surgery, Semmelweis University, Budapest, Hungary; e Faith and Health Division, Methodist Le Bonheur Healthcare, Memphis, TN, USA; f Division of Health Systems Management and Policy, School of Public Health, The University of Memphis, Memphis, TN, USA; g Transplant Immunology Laboratory, DCI Inc, Nashville, TN, USA; h Sandra Atlas Bass Center for Liver Diseases and Transplantation, Department of Medicine, Northshore University Hospital/Northwell Health, Manhasset, NY, USA; i Division of Nephrology, Department of Medicine, University of Tennessee Health Science Center, Memphis, TN, USA

**Keywords:** Donor specific antibody, DSA, *de novo* DSA, simultaneous liver–kidney transplantation, length of hospital stay, hospitalization

## Abstract

**Background:**

*De novo* Donor Specific Antibodies (DSA) are considered as a risk factor for the kidney allograft outcomes in recipients after simultaneous liver–kidney transplantation (SLKT). We hypothesized that length of hospital stay (LOS) might be associated with *de novo* DSA development of due to the increased likelihood of receiving blood transfusions with reduced immunosuppressive regimens.

**Methods:**

This study is a single-center, retrospective cohort study consisting of 85 recipients who underwent SLKT from 2009 to 2018 in our hospital. We divided the patients into two groups according to LOS [long hospital stay (L) group (LOS >14 days) and short hospital stay (S) group (LOS ≤14 days)]. Propensity score (PS) has been created using logistic regression to predict LOS greater than median of 14 days. The association between the presence of *de novo* DSA and LOS was assessed by logistic regression models adjusted for PS.

**Results:**

The mean age at transplantation of the entire cohort was 55.5 ± 10.1 years. Sixty percent of the recipients were male and Caucasian. Median LOS in (L) group was three-fold longer than (S) group [L: median 30 days (IQR: 21–52), S: median 8.5 days (IQR: 7–11)]. Eight patients developed *de novo* DSA after SLKT (9.4%), all of them were in (L) group. Longer LOS was significantly associated with higher risk of development of *de novo* DSA in unadjusted (OR_+ each 5 days_: 1.09, 95% CI:1.02–1.16) and PS adjusted (OR_+ each 5 days_: 1.11, 95% CI:1.02–1.21) analysis.

**Conclusion:**

Longer hospitalization is significantly associated with the development of *de novo* DSA in SLKT.

## Introduction

Post-transplant donor-specific antibodies (DSA), either identified pre-transplant (persistent DSA) or newly developed (*de novo* DSA) beyond the absorptive capacity conferred by allograft liver [1–4], present a risk factor for patient- and allograft kidney outcome after simultaneous liver–kidney transplantation (SLKT) [5,6]. While the majority of pre-transplant DSA become undetectable after liver transplantation alone (LTA) [7] and after SLKT [8,9], about 10–20% of recipients develop *de novo* DSA after LTA and SLKT [5,6,10]. Currently, the risk factors associated with newly developed *de novo* DSA have not been well investigated in SLKT. The identification of potentially modifiable risk factors influencing *de novo* DSA development after SLKT might have positive effects on patient and graft survival.

Length of hospital stay (LOS) after surgery is one of the relevant clinical outcomes measured in many clinical settings [11–13]. Longer LOS has been shown to be associated with patient characteristics such as age, higher morbidity, worsened frailty, increased number and severity of comorbidities and unfavorable clinical outcomes and complications [11–16]. Previous studies also showed longer LOS was associated with more infectious complications; which could lead to decreased use of immunosuppressive medications or larger amount of blood product transfusions [14,15,17].

Infectious complications and blood transfusions have also been identified as risk factors for longer LOS in liver transplant recipients [18–20]. Infectious complications can cause cessation or reduction of immunosuppressive medications; while blood transfusions can cause allo-sensitization [21,22]. Furthermore, early allograft liver dysfunction (EAD) was also identified as a risk factor for longer LOS [23]. EAD grafts may lose the capacity to fully absorb existing pre-transplant DSA, which might lead to persistent DSA after SLKT. Longer hospital stay might serve as a surrogate marker for these sensitization events, in addition to demonstrating association with *de novo* DSA development after SLKT.

In this retrospective study, we hypothesized that LOS is associated with a higher probability of *de novo* DSA development after SLKT. We evaluated the association between LOS and *de novo* DSA development using a single-center cohort in the modern immunosuppressant era.

## Materials and methods

### Cohort definition and data source

This is a single-center, retrospective cohort study. We enrolled 85 consecutive recipients who underwent SLKT from 1 April 2009 to 28 February 2018 at Methodist University Hospital in Memphis, TN, USA. Exclusion criteria being those who were less than 18 years old or equal, but no patients were excluded from this study. Any information from recipients or deceased donors, as well as immunologic information were extracted from local electronic medical record (EMR), from the UNOS database, and from our HLA laboratory database until February 9th, 2019. We captured all data into a Research Electronic Data Capture (REDCap) system, which is an electronic data capturing tool hosted at the Center for Biomedical Informatics, the University of Tennessee Health Science Center [24]. REDCap (Research Electronic Data Capture) is a secure, web-based application designed to support data capture for research studies, providing: 1) an intuitive interface for validated data entry; 2) audit trails for tracking data manipulation and export procedures; 3) automated export procedures for seamless data downloads to common statistical packages; and 4) procedures for importing data from external sources.

The clinical and research activities being reported are consistent with the Principles of the Declaration of Istanbul as outlined in the ‘Declaration of Istanbul on Organ Trafficking and Transplant Tourism’. All deceased donated organs were procured based on SLKT allocation policy in Organ Procurement and Transplantation Network (OPTN)/United Network for Organ Sharing (UNOS) [25] and thus no organs were procured from prisoners [26].

This study was approved by the Institutional Review Committee of The University of Tennessee Health Science Center (18-06146-XP). This is retrospective observational data collection and waiver for the consent form from participated recipients was approved by our IRB. Furthermore, this study did not need the consent from deceased donor either since this was not an interventional study [27].

### Immunosuppression protocol

The applied immunosuppression protocol was similar for all patients regardless of pre-transplant sensitization status [28]. As induction therapy, all patients received intravenous methylprednisolone (500 mg) on day 0, and rabbit anti-thymocyte globulin (1.5 mg/kg) on day 0 and again on post-operative day 2. Mycophenolate mofetil (MMF) or equivalent mycophenolic acid was started immediately post-operatively and continued until month three. Tacrolimus was started after improvement in kidney function, usually between post-operative day 3–7, and target trough range was 6–8 ng/mL until 3 months post-transplantation and 3–5 ng/mL thereafter. No patients received pre-transplant desensitization before SLKT. All patients were maintained on a steroid-free protocol.

### Exposure

The LOS was defined as the exposure in this study. LOS was calculated as sum of the days from date of admission to date of discharge. LOS was divided into two groups according to the median of 14 days. Long hospital stay group was defined as LOS greater than 14 days [(L) group] and short hospital stay group was defined as LOS less than 14 days or equal [(S) group]. For the sensitivity analysis, the threshold of long LOS was replaced with 28 days, which was two-times longer compared to the main analysis and based on the previous literature [29,30].

### Covariates

We extracted data about recipients’ baseline characteristics including age, gender, race, body mass index (BMI), marital status, insurance, cause of end-stage liver disease (ESLD), cause of chronic kidney disease (CKD)/end stage renal disease (ESRD), pre-SLKT dialysis information including length and type (maintenance dialysis was defined as dialysis for ≥3 months; acute dialysis initiation was defined as dialysis for <6 weeks; while sub-acute dialysis was defined as dialysis for ≥6 weeks-<3 months before transplantation), comorbid conditions (diabetes: DM and hypertension: HTN), the Model for End-stage Liver Disease (MELD) score at SLKT, the number of Human Leucocyte Antigen (HLA) mismatches, calculated panel reactive antibody (cPRA), and cold-ischemic time (CIT) of donated kidney from the above mentioned sources. As post-SLKT information, first discharge destination, delayed allograft kidney function (DGF), primary non-function (PNF) on allograft kidney, death following transplant hospitalization. DGF was defined as needs of dialysis within one-week post SLKT [31] and PNF was defined as the condition on dialysis dependence after SLKT. Deceased donors’ information included age, sex, race, cause of death, history of hypertension and diabetes, and expanded criteria donor (ECD) status were also collected. All donations occurred after brain death. There was only one recipient with missing value of CIT, thirteen recipients had missing value of cPRA, and one recipient had missing value of HLA mismatches. These missing values were not imputed.

### Outcomes

Primary outcome was incidence of development of *de novo* DSA after SLKT. Furthermore, prevalence of post-transplant DSA (persistent or *de novo* DSA), was also assessed as secondary outcome. Persistent DSA was defined as DSA detected before and after SLKT, while *de novo* DSA was defined as newly developed DSA following SLKT. We assessed the incidence of persistent DSA or *de novo* DSA or in each patient after SLKT. HLA specificities were identified using a solid phase single antigen bead platform (SAB; One Lambda Inc, a division of Thermo-Fisher, Canoga Park, CA, USA) combined with Luminex xMAP technology (Luminex Corporation., Northbrook, IL, USA). Patients with any observed class DSA were categorized as *de novo* DSA (+), while those negative for identified *de novo* DSA were classified *de novo* DSA (−). We defined post-DSA that has a mean fluorescence intensity (MFI) value that is elevated compared to pre-transplant levels or has an MFI of greater than 1000. The median days of measurement of DSA from SLKT in both any post-transplant DSA and *de novo* DSA were also calculated as sum of days. The DSA measurements were performed as per clinical indication. The methodology of detecting DSA including the decision of measurements, technical issues, and thresholds of DSA was not changed during the study period.

### Statistical analysis

Baseline characteristics were described for the entire cohort and for groups categorized based on the LOS and presented as mean ± standard deviation (SD) or median and interquartile range (IQR) for continuous variables and percent for categorical variables as appropriate. Differences between groups were assessed by student *T*-test or Mann-Whitney test for continuous variables and chi-square-test (or Fisher’s exact test) for categorical variables.

We calculated the propensity score (PS) for probability of long hospital stay in the main analysis (LOS >14 days versus ≤14 days) and in the sensitivity analysis (LOS >28 days versus ≤28 days) using logistic regression model (presented in Table S1, Supplementary material), including all available covariates without missing values for LOS, including age, gender, race, marital status, insurance, cause of ESLD, diabetes, hypertension, BMI, blood type, MELD score, first discharge destination, and DGF. The purpose of this step was to be able to adjust for co-variates which showed association with LOS. Because only 8 patients developed *de novo* DSA, we were able to adjust for only one variable in our adjusted logistic regression analysis used for assessing association between LOS (as exposure) and development of *de novo* DSA or any post-transplant DSA (as outcomes). We then assessed logistic regression analysis, unadjusted and PS score adjusted, to calculate the relative risk (odds ratio) for newly developing *de novo* DSA or any post-transplant DSA.

*p* values were two-sided and significance level was set at less than .05 for all analyses. All analyses were conducted using STATA Version 13 (STATA Corporation, College Station, TX).

## Results

### Baseline characteristics

Table 1 shows the baseline characteristics of the entire cohort for both (L) and (S) groups. The median age at SLKT was 55.5 ± 10.1 years old and approximately 60% of the patients were Caucasian males. The leading causes of ESLD were hepatitis C and alcoholic hepatitis. The patients in (L) group had significantly higher MELD scores, prevalence of acute dialysis initiation shorter length of dialysis before SLKT (maintenance and sub-acute dialysis) and higher prevalence of alcoholic hepatitis as a cause of ESLD compared with (S) group patients.

**Table 1.** Baseline characteristics of the entire cohort and divided by long and short hospital stay groups.

Baseline characteristics	Entire cohort, *N* = 85	Long hospital stay group (L), *N* = 43	Short hospital stay group (S), *N* = 42	*p* Value*
Recipient information				
Age, years, mean ± SD	55.5 ± 10.1	56.7 ± 9.4	54.4 ± 10.7	.292
Gender, male, *n* (%)	53 (62.4)	28 (65.1)	25 (59.5)	.595
BMI, kg/m^2^, mean ± SD	27.0 ± 6.4	28.2 ± 7.2	28.6 ± 5.6	.827
Race, *n* (%)				.866
African American	22 (25.9)	11 (25.6)	11 (26.2)	
Caucasian	51 (60.0)	26 (60.5)	25 (59.5)	
Other	12 (14.1)	6 (14.0)	6 (14.3)	
Marital status, Married, *n* (%)	52 (61.2)	27 (62.8)	25 (59.5)	.783
Insurance, *n* (%)				.680
Private	31 (36.5)	16 (37.2)	15 (35.7)	
Medicaid	6 (7.1)	4 (9.3)	2 (4.8)	
Medicare	48 (56.5)	23 (53.5)	25 (59.5)	
Presence of preexisting CKD, *n* (%)	74 (87.1)	32 (74.4)	42 (100)	<.001
Cause of CKD, *n* (%)				.647
Hypertension	8/74 (10.8)	4/32 (12.5)	4/42 (9.5)	
Diabetes	14/74 (18.9)	6/32 (18.8)	8/42 (19.0)	
Glomerulonephritis	4/74 (5.4)	2/32 (6.3)	2/42 (4.8)	
Cystic disease	3/74 (4.1)	0	3/42 (7.1)	
Metabolic/inherited disease	2/74 (2.7)	0	2/42 (4.8)	
Other/unknown	43/74 (58.1)	20/32 (62.5)	23/42 (54.8)	
Dialysis status before SLKT, *n* (%)				.003
Maintenance dialysis, *n* (%)	38 (44.7)	19 (44.2)	19 (45.2)	
Sub-acute dialysis, *n* (%)	9 (10.6)	6 (14.0)	3 (7.1)	
Acute dialysis initiation before SLKT, *n* (%)	16 (18.8)	13 (30.2)	3 (7.1)	
Length of dialysis before SLKT (maintenance and sub-acute dialysis group), months, median (IQR)	8.9 (3.9, 36.8)	7.1 (3.3, 20.4)	13.0 (7.4, 44.6)	.077
Length of acute dialysis before SLKT (acute dialysis group only), days, median (IQR)	13.5 (6.5, 24.5)	12.0 (8.0, 24.0)	19.0 (5.0, 27.0)	.638
Cause of ESKD (maintenance and sub-acute group), *n* (%)				.278
Acute on CKD,	12/47 (25.5)	8/25 (32.0)	4/22 (18.2)	
Same as CKD	35/47 (74.5)	17/25 (68.0)	18/22 (81.8)	
Cause of ESLD, *n* (%)				.127
HCV	24 (28.2)	7 (16.3)	17 (40.5)	
Alcoholic hepatitis	22 (25.9)	14 (32.6)	8 (19.0)	
HCV and Alcoholic hepatitis	3 (3.5)	1 (2.3)	2 (4.8)	
NASH	16 (18.8)	9 (20.9)	7 (16.7)	
Other	20 (23.5)	12 (27.9)	8 (19.0)	
Comorbidity – diabetes, *n* (%)	35 (41.2)	18 (41.9)	17 (40.5)	.897
Comorbidity – hypertension, *n* (%)	61 (71.8)	29 (67.4)	32 (76.2)	.370
HLA mismatches locus A, *n*, mean ± SD	1.6 ± 0.6	1.5 ± 0.7	1.5 ± 0.6	.859
HLA mismatches locus B, *n*, mean ± SD	1.7 ± 0.5	1.7 ± 0.5	1.7 ± 0.5	.820
HLA mismatches locus DR, *n*, mean ± SD	1.5 ± 0.5	1.5 ± 0.6	1.6 ± 0.5	.535
Total HLA mismatches, *n*, mean ± SD	4.8 ± 1.0	4.8 ± 1.1	4.9 ± 0.9	.750
cPRA, %, median (IQR)	0 (0, 8)	0 (0, 3.0)	0 (0, 13.0)	.866
Cold ischemic time of donated kidney, minutes, mean ± SD	496.5 ± 114.6	514.3 ± 114.1	478.6 ± 113.6	.155
MELD score, mean ± SD	28.3 ± 6.5	31.7 ± 6.3	24.9 ± 4.6	<.001
Donor information				
Age, years, mean ± SD	28.9 ± 11.1	29.2 ± 10.9	28.6 ± 11.4	.793
Gender, male, *n* (%)	47 (55.3)	27 (62.8)	20 (47.6)	.160
Donor Race, *n* (%)				.163
Caucasian	65 (76.5)	36 (83.7)	29 (69.0)	
African American	18 (21.2)	7 (16.3)	11 (26.2)	
Hispanic	2 (2.4)	0	2 (4.8	
Donation after brain death, *n* (%)	85 (100)	43 (100)	42 (100)	–
Cause of death, *n* (%)				.785
Anoxia	27 (31.8)	14 (32.6)	13 (31.0)	
Cerebrovascular/stroke	18 (21.2)	8 (18.6)	10 (23.8)	
Head trauma	33 (38.8)	18 (41.9)	15 (35.7)	
Central nerve system tumor	1 (1.2)	0	1 (2.4)	
Other	5 (5.9)	2 (4.7)	3 (7.1)	
Comorbidity-diabetes, *n* (%)	1 (1.2)	1 (2.3)	0	.320
Comorbidity-hypertension, *n* (%)	11 (12.9)	7 (16.3)	4 (9.5)	.354
Expanded criteria donor, *n* (%)	1 (1.2)	1 (2.3)	0	.320

BMI: Body mass index; CKD: Chronic kidney disease; cPRA: calculated panel reactive antibody; ESKD: End-stage kidney disease; ESLD: End-stage liver disease; HCV: Hepatitis C; HLA: Human Leukocyte Antigen; IQR: Interquartile range; MELD: Model of end-stage liver disease; NASH: Nonalcoholic steatohepatitis; SLKT: Simultaneous liver–kidney transplantation.

^*^ Compared between Long hospital stay group (L) group and short hospital stay group (L) groups. *p* values for continuous variables with mean ± SD are result of *t*-test and with median (IQR) are result of Mann–Whitney test, and categorical variables are chi-square test.

### Length of hospital stay and post-transplant events

Table 2 shows post-SLKT information of LOS, first discharge destination, and incidence of outcomes, DGF, PNF, and death following SLKT hospitalization. The median LOS of entire cohort was 15 days (IQR: 9–30 days) and duration from transplantation to discharge was median 11 days (IQR: 8–21 days). The median LOS in (L) group was significantly longer than that in (S) group [30 days (IQR: 21–52 days) and 8.5 days (IQR: 7–11 days), *p* < .001], respectively. In (L) group, incidence of returning to home as first discharge destination was significantly lower than in (S) group (27.9% and 69.0%, respectively, *p* = .001). No PNF was observed; however, DGF of the allograft kidney was significantly higher in (L) group. Incidence of death during SLKT hospitalization was higher in (L) group compared to (S) group.

**Table 2.** Post-transplant characteristics of the entire cohort and divided by long and short hospital stay groups.

	Entire cohort, *N* = 85	Long hospital stay group, (L) *N* = 43	Short hospital stay group (S), *N* = 42	*p* Value*
Length of stay for SLKT admission, days, me\dian (IQR)	15.0 (9.0, 30.0)	30.0 (21.0, 52.0)	8.5 (7.0, 11.0)	<.001
Duration from admission to transplantation, days, median (IQR)	1.0 (1.0, 6.0)	6.0 (1.0, 16.0)	0 (0, 1.0)	<.001
Duration from transplantation to discharge, days, median (IQR)	11.0 (8.0, 21.0)	21.0 (12.0, 42.0)	8.0 (6.0, 10.0)	<.001
Delayed graft function (kidney), *n* (%)	15 (17.6)	12 (27.9)	3 (7.1)	.012
Primary non-function (kidney), *n* (%)	0	0	0	–
Death during hospitalization, *n* (%)	9 (10.6)	7 (16.3)	2 (4.8)	.084
First discharge destination, *n* (%)				.001
Home	41 (48.2)	12 (27.9)	29 (69.0)	
Home and taking health service	22 (25.9)	13 (30.2)	9 (21.4)	
Rehabilitation hospital	12 (14.1)	10 (23.3)	2 (4.8)	
Others	10 (11.8)	8 (18.6)	2 (4.8)	
Incidence of developing of *de novo* DSA, n (%)	8 (9.4)	8 (18.6)	0	.003
Duration from transplant to measurement of *de novo* DSA, days, median (IQR)	53.0 (14.0, 280.5)	53.0 (14.0, 280.5)	N/A	–
Prevalence of any post-transplant DSA, n (%)	12 (14.1)	11 (25.6)	1 (2.4)	.002

DSA: Donor specific antibody; IQR: Interquartile range; SLKT: Simultaneous liver–kidney transplantation.

*Compared between long hospital stay and short hospital stay groups. *p* values for continuous variables with median (IQR) are result of Mann–Whitney test and categorical variables are chi-square test.

### Developing of de novo DSA and prevalence of any post-transplant DSA

**Figure 1.**
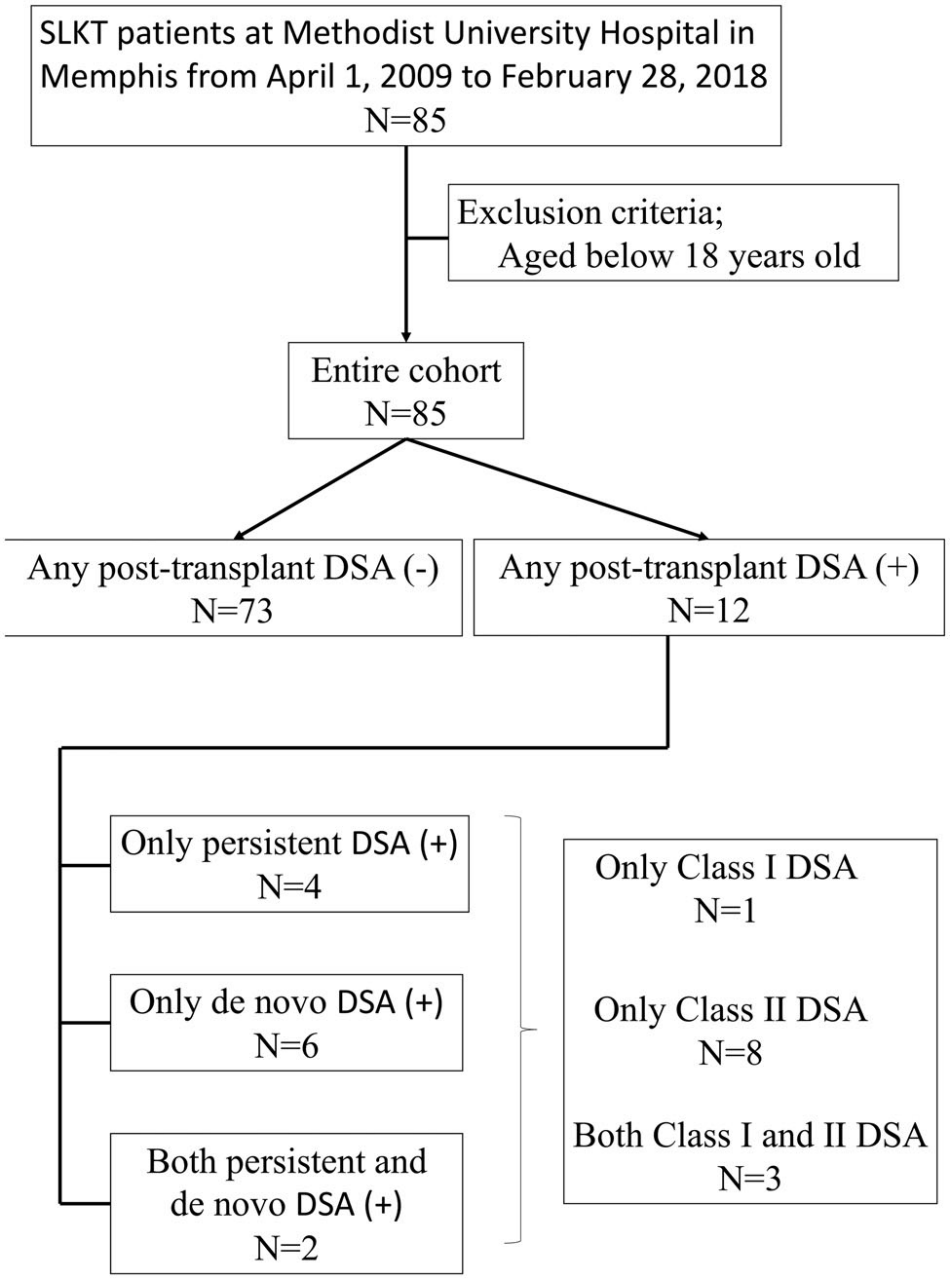
Flow chart of patient selection and incidence and prevalence of *de novo* DSA and persistent DSA. Abbreviations: DSA: Donor-specific antibody; *de novo* DSA: newly developed DSA; N: Number; SLKT: Simultaneous liver–kidney transplantation.

Eight patients developed *de novo* DSA (9.4%) and 12 patients were identified with any post-transplant DSA consisted of both persistent and *de novo* DSA (14.1%) after SLKT (Figure 1). All patients who developed *de novo* DSA and 11 out of 12 patients with any post-transplant DSA were in (L) group. The median days from SLKT was 22.0 days (IQR: 8.0–280.5 days) for measurement of any post-transplant DSA and 53.0 days was (IQR: 14.0–280.5 days) for measurement of *de novo* DSA (Table 2).

### Probability of developing de novo DSA and any post-transplant DSA

Longer LOS was significantly associated with higher risk of development of *de novo* DSA in unadjusted (OR_+ each 5 days_: 1.09, 95% CI: 1.02–1.16) and PS adjusted (OR_+ each 5 days_: 1.11, 95% CI: 1.02–1.21) analysis (Table 3). Longer LOS was also significantly associated with higher risk of prevalence of any post-transplant DSA consisted of both persistent and *de novo* DSA in unadjusted (OR_+ each 5 days_: 1.08, 95% CI: 1.02–1.15) and PS adjusted (OR_+ each 5 days_: 1.08, 95% CI: 1.01–1.16) analysis (Table 3).

**Table 3.** Probability of development of de-novo DSA and any post-transplant DSA using unadjusted and propensity score adjusted logistic regression models.

	Risk of *de novo* DSA			Risk of any post-transplant DSA		
	Odds ratio	95% CI	*p* value	Odds ratio	95% CI	*p* value
Un-adjusted						
Length of hospital stay (each 5 days)	1.09	1.02–1.16	.010	1.08	1.02–1.15	.011
Adjusted						
Length of hospital stay (each 5 days)	1.11	1.02–1.21	.014	1.08	1.01–1.16	.028
PS score	0.27	0.02–4.13	.343	1.08	0.13–8.68	.944

DSA: Donor specific antibody; PS: Propensity score; 95% CI: 95% confidence interval.

### Probability of developing de novo DSA and any post-transplant DSA in the sensitivity analysis

Longer LOS defined as >28 days was significantly associated with higher risk of development of *de novo* DSA and prevalence of any post-transplant DSA consisted of both persistent and *de novo* DSA in unadjusted and PS adjusted analysis (Table S2, Supplementary material).

## Discussion

In this single center, retrospective study, we found significant associations between longer hospitalization and higher probability of both persistent post-transplant DSA and *de novo* DSA development after SLKT. In addition, our study indicates that longer LOS occurred more frequently in SLKT patients with higher MELD scores and higher incidence of DGF in allograft kidney. Furthermore, 30% of the patients in (L) group, compared to exact percentage in (S) group, were discharged to their home after SLKT. All of those who developed *de novo* DSA had longer hospitalizations. Our study implies that LOS might be a useful surrogate marker of a higher probability for persistent DSA and *de novo* DSA development. Patients, if not all, with longer hospitalization should be routinely screened for DSA after SLKT. We believe this is the first report to evaluate an association between the length of hospitalization and post-transplant DSA development in SLKT.

Sensitized status in SLKT has tended to be neglected due to the absorptive capacity by allograft liver [1–4]. In fact, sensitization before SLKT is not a contraindication of transplantation from several clinical practice guidelines [32–34]. However, post-transplant DSA, especially *de novo* ClassII DSA, has been thought to be a significant risk factor for patient-, allograft liver-, and allograft kidney outcome [5]. No previous study has assessed the risk factors of developing *de novo* DSA in SLKT, although the potential for exposure to sensitizing events might be expected to be higher in SLKT than liver or kidney transplantation alone (LTA and KTA). Although LTA patients typically do not undergo maintenance dialysis therapy before LTA, 30–50% of SLKT candidates undergo dialysis therapy immediately prior to SLKT [35,36]. We observed this finding in our cohort as well. Simultaneous liver–kidney transplant patients could be more fragile prior to transplantation and require more blood transfusions peri-operatively compared to KTA patients [37]. Higher prevalence of maintenance dialysis therapy and history of blood transfusions in SLKT patients has been associated with sensitization [38–40], which leads to higher probability of sensitization before and after transplantation compared to LTA and KTA patients.

We constructed a conceptual model of the assessed relationship between LOS and DSA. We identified an association between longer LOS and developing *de novo* DSA. The relationship between assumptive exposures, (blood transfusions, infectious events) and EAD and LOS has already been reported in other patient cohorts [14,15] as well as recipients with LTA [18–20,23]. We wanted to clarify a direct relationship between presumptive exposures and *de novo* DSA development. Our preliminary findings suggest LOS might be a consequence of DGF on allograft kidney secondary to persistent DSA or *de novo* DSA development or other potential intermediated mediators (indicator). Although we could not identify the exposure in each patient, a relationship between presumptive exposures and *de novo* DSA development could exist in accordance with this conceptual model.

Longer hospitalization also showed significant association with a higher probability of persistent or *de novo* post-transplant DSA. In our cohort, 4 patients had persistent DSA; 2 patients had both persistent and *de novo* DSA after SLKT (Figure 1). Five of the six patients with persistent DSA after SLKT, were in (L) group. Our results suggest patients with pre-transplant DSA and longer LOS should be routinely monitored for post-transplant DSA.

Several limitations should be noted with this study. Although this is one of the largest SLKT cohorts studied to date, our patient group was still small and had a relatively low number of events. Because this was a retrospective cohort study, we could not conclude causal relationship between LOS and developing *de novo* DSA despite the finding the median days of measurement of DSA after SLKT was almost double compared to length of hospital stay in (L) group. Furthermore, DSA measurement was indication based and not routine, which might have caused observational bias. In this study, we were not able to assess the pathophysiological role of Class I and II DSA separately, as almost all of the patients with post-transplant DSA had at least Class II DSA (*N* = 11/12) (Figure 1). Finally, the observed differences in clinical practices observed across different transplant programs may lead to different definitions of LOS. These differences could be the generalizability of our findings. In fact, albeit LOS was replaced with 28 days or longer in our sensitivity analysis, LOS was still significant risk factor for the developing of *de novo* DSA and prevalence of any post-transplant DSA. Additional prospective and larger studies that include protocol DSA measurement are highly warranted.

Despite some limitations, our study has confirmed several previous findings. LOS has been shown to be predicted by factors such as ethnicity, discharge destination, and type of insurance [13]. In our study, (L) group patients were more likely to be discharged to rehabilitation or discharged with home service than (S) group. Despite have low event numbers, we were able to adjust for these variables using a PS score. This is the first report to identify LOS as a risk factor for *de novo* DSA development in SLKT. Our results support the conclusion that DSA monitoring can be implemented into clinical practice for SLKT patients.

In conclusion, longer hospitalization was significantly associated with higher probability of persistent DSA and *de novo* DSA development after SLKT. Although longer hospitalization could be an indicator and not a direct cause for *de novo* DSA development, DSA monitoring after LOS might be able to help providers improve outcomes after SLKT. Additional prospective and larger studies are highly warranted to identify more modifiable and non-modifiable predictors for persistent and *de novo* DSA development following SLKT.

## Supplementary Material

Supplemental Material

## Data Availability

The datasets generated during the current study are available from the corresponding author on reasonable request.
