# Supplementary material for: Association between longer hospitalization and development of de novo donor specific antibodies in simultaneous liver–kidney transplant recipients
Source: Ren Fail. 2019 Dec 26;42(1):40–7. doi: 10.1080/0886022X.2019.1705338 (PMC6968335; doi:10.1080/0886022X.2019.1705338)
Supplement: Supplemental Material [file IRNF_A_1705338_SM2221.pdf]

Association between Longer Hospitalization and Development of de novo Donor Specific Antibodies in Simultaneous Liver-Kidney Transplant Recipients

Masahiko Yazawa, MD, PhD[1,2,3], **Orsolya Cseprekal,** MD, PhD[1,2,4], **Ryan A. Helmick,** MD[1,2], **Manish Talwar,** MD[1,2], **Vasanthi Balaraman,** MD[1,2], **Pradeep S. B. Podila,** PhD, MS, MHA[5,6], **Sallyanne Fossey,** PhD, D(ABHI)[7], **Sanjaya K. Satapathy,** MD, MS[8], **James D. Eason,** MD[1,2]**, Miklos Z. Molnar*,** MD, PhD[1,2,4,9]

***Corresponding author:**

Miklos Z Molnar, M.D., Ph.D., FEBTM, FERA, FASN

Associate Professor of Medicine, University of Tennessee Health Science Center

James D. Eason Transplant Institute, Methodist University Hospital

Methodist Transplant Epidemiology Research Group

1211 Union Ave, Memphis, TN, United States, 38104

Phone: 1-901-516-0751

Fax: 1-901-516-8994

E-mail: mzmolnar@uthsc.edu

Supplementary Information

Supplementary Table 1. Probability of long hospital stay in main analysis (>14 days versus ≤14 days) and in sensitivity analysis (>28 days versus ≤28 days) using logistic regression analysis

	Main analysis (>14 days)			Sensitivity analysis (>28 days)		
**Covariates**	**Odds ratio**	**95% CI**	***p* value**	**Odds ratio**	**95% CI**	***p* value**
**Recipient information**						
Age (+1 year)	1.11	1.01 to 1.23	0.029	1.44	0.91 to 2.27	0.120
Gender (vs male)	0.57	0.08 to 4.05	0.570	0.10	0.001 to 7.99	0.308
BMI (+ 1 unit: kg/m^2)	0.76	0.60 to 0.95	0.018	1.19	0.81 to 1.77	0.373
Race						
Caucasian (vs African American)	0.35	0.02 to 5.61	0.458	0.06	<0.001 to 10.1	0.280
Other (vs African American)	0.45	0.02 to 8.79	0.599	3.94	0.02 to 1022.5	0.629
Marital status married (vs other)	1.32	0.23 to 7.43	0.755	0.41	0.007 to 22.7	0.665
Insurance						
Medicaid (vs. private)	25.8	0.09 to 7388.5	0.260	<0.001	<0.00001 to >10,000,000	0.457
Medicare (vs. private)	0.28	0.03 to 2.52	0.255	0.007	<0.001 to 14.7	0.202
Cause of ESLD	2.355	1.287 to 4.309	0.005			
Alcoholic hepatitis (vs. HCV)	47.8	1.90 to 1202.5	0.019	360.5	0.7 to 185498.8	0.065

HCV and Alcoholic hepatitis (vs. HCV)	315.7	1.33 to 74871.4	0.039	N/A	N/A	N/A
NASH (vs. HCV)	51.7	0.86 to 3094.9	0.059	75.8	0.41 to 13941.3	0.104
Other (vs. HCV)	1099.1	12.6 to 96162.7	0.002	361094.5	0.34 to >10,000,000	0.070
Comorbidity – diabetes (vs. absence)	4.26	0.51to 35.7	0.182	123.1	0.51 to 29580.5	0.085
Comorbidity – hypertension (vs. absence)	12.4	0.69 to 223.7	0.088	1954001	0.41 to >10,000,000	0.065
MELD score (+1 score)	1.54	1.18 to 2.01	0.001	1.77	0.99 to 3.17	0.055
First discharge destination						
Home with health service (vs. home without health service)	0.72	0.09 to 5.89	0.762	0.27	<0.001 to 95.1	0.665
Rehabilitation hospital (vs. home without health service)	261.2	8.02 to 8509.3	0.002	85230.4	1.14 to >10,000,000	0.047
Other (vs. home without health service)	39.4	1.47 to 1062.0	0.029	110966.8	1.66 to >10,000,000	0.040
Delayed graft function on allograft kidney (vs. absence)	80.5	2.80 to 2312.3	0.010	3422.1	0.14 to >1,000,000	0.114
Blood type						
Type A (vs. type O)	0.41	0.05 to 3.25	0.400	0.02	<0.001 to 7.07	0.199
Type B (vs. type O)	0.006	0.0001 to 0.26	0.008	<0.001	<0.00001 to 27.5	0.134
Type AB (vs. type O)	9.92	<0.00001 to >10,000,000	0.750	42364.2	1.06 to >10,000,000	0.049

Supplementary Table 2. Probability of development of de-novo DSA and any post-transplant DSA using unadjusted and propensity score adjusted logistic regression models in sensitivity analysis.

	Risk of de novo DSA			Risk of any post-transplant DSA		
	Odds ratio	95% CI	*p* value	Odds ratio	95% CI	*p* value
Un-adjusted						
Length of hospital stay (each 5 days)	1.09	1.02-1.16	0.010	1.08	1.02-1.15	0.011
Adjusted						
Length of hospital stay (each 5 days)	1.15	1.02-1.28	0.017	1.11	1.01-1.21	0.022
PS score	0.07	0.001-4.00	0.200	0.30	0.18-5.16	0.408

Abbreviations: DSA: Donor specific antibody; PS: Propensity score; 95% CI: 95% confidence interval
